# Overview of the Implementation of the First Year of Immunization against Human Papillomavirus across Different Administrative Units in Serbia and Montenegro

**DOI:** 10.3390/vaccines12070803

**Published:** 2024-07-19

**Authors:** Mirjana Štrbac, Milko Joksimović, Vladimir Vuković, Mioljub Ristić, Goranka Lončarević, Milena Kanazir, Nataša Nikolić, Tatjana Pustahija, Smiljana Rajčević, Stefan Ljubičić, Marko Koprivica, Dragan Laušević, Vladimir Petrović

**Affiliations:** 1 Institute of Public Health of Vojvodina, Futoška 121, 21000 Novi Sad, Serbia; mioljub.ristic@izjzv.org.rs (M.R.);; 2 Institute of Public Health of Montenegro, Džona Džeksona bb, 81110 Podgorica, Montenegro; 3 Faculty of Medicine, University of Novi Sad, Hajduk Veljkova 3, 21000 Novi Sad, Serbia; 4 Institute of Public Health of Serbia “Dr Milan Jovanović Batut”, 11000 Belgrade, Serbia

**Keywords:** human papillomavirus, HPV vaccine, prevention, oncology, Serbia, Montenegro

## Abstract

Despite the availability of a safe and effective vaccination, uptake of human papillomavirus (HPV) vaccination remains low worldwide. We aimed to analyze the coverage of HPV immunization during the first year of the immunization program and the sociodemographic characteristics across different administrative units in Serbia and Montenegro. Coverage of HPV vaccination in Serbia for females aged 9–14 and 15–19 years was 5.5% and 5.9%, respectively. The coverage rate of immunization against HPV in Montenegro for girls aged 9–14 years was 22.1%. Within Serbia, only one administrative region (Moravica) had HPV immunization coverage in girls 9–19 years old above 10%, 11 districts had coverage from 5 to 10%, while 13 districts had coverage below 5%. As per Montenegro, two administrative units, Cetinje and Berane, reported the highest coverage, with 39% and 36.4% of vaccinated eligible girls, respectively. When we explored the coverage of HPV immunization among girls aged 9–19 years across different regions in Serbia, we observed that the level of coverage did not correlate with the number of pediatricians or with the population density. In Montenegro, we observed a similar situation. On the other hand, we noticed a statistically significant moderate negative correlation (r = −0.446; *p* = 0.026) between HPV immunization coverage and the percentage of illiterate women in the administrative units. Comparing the coverage between the two countries we found that the higher coverage in Montenegro corresponded with a smaller number of female populations aged 9–14 years, with higher average net monthly income, with smaller population density and smaller number of pediatricians, among divorced persons, and among those without formal education or incompletely primary education. Taking into account the experiences in Montenegro, increasing immunization coverage in Serbia could be achieved through a more vigorous educational campaign targeting schools, the general population, and healthcare workers as well as by additionally incentivizing those engaged in these activities.

## 1. Introduction

Cervical cancer is the 10th leading cause of female cancer and the third most common cancer in women aged 15 to 44 years in Europe, but in Serbia, it is the fifth most common female cancer overall and the second most common in the age group of 15–44 years [1]. Montenegro, on the other hand, has the highest rate of cervical cancer in Europe, according to WHO’s estimates from 2020 the age-standardized incidence rate is 26.2/100,000 women and 10.5 women died from the disease for every 100,000 women in the country [2]. Regarding the geographical world regions, the incidence and mortality of cervical cancer vary. They are higher in underdeveloped areas (e.g., Sub-Saharan African and Southeastern Asian countries), which can be explained by limited healthcare infrastructure, lack of screening programs, and minimal access to HPV vaccination, contributing to very high incidence rates of cervical cancer (ASRI > 26.26/100,000). Contrary to that, economically developed countries (e.g., Western Europe) have a low burden of cervical cancer. These countries typically have robust healthcare systems with widespread access to screening programs (such as Pap smears) and HPV vaccination. Early detection and treatment options are advanced, leading to lower incidence rates and improved survival rates among cervical cancer patients (ASRI < 7.56/100,000). Somewhere in between, developing countries, such as Serbia and Montenegro, have a moderate to high burden of incidence and mortality of cervical cancer (ASRI 17.10–26.26/100,000). In these countries, healthcare resources may be limited, leading to challenges in implementing comprehensive cervical cancer prevention and control measures. Screening programs may be sporadic, and access to HPV vaccination could be limited, especially in rural or underserved areas [3].

Persistent infection with an oncogenic strain of the human papillomavirus (HPV) is considered a necessary factor for the formation of precancerous lesions and the occurrence of cervical cancer. In fact, oncogenic strains of the HPV, of which type 16 and type 18 are most common, cause almost 100% of cases of cervical cancer, along with 90% of anal, 70% of vaginal, 40% of vulvar, 50% of penile and 13% to 72% of oropharyngeal cancers [4]. Considering that HPV prevalence and genotype distribution of normal and abnormal cervical lesions are missing in the updated IARC Human Papillomavirus and Related Diseases Report for Serbia and Montenegro, only recent publications can provide insight into data on the prevalence of HPV-infected women. According to them, the HPV infection rate in Serbian and Montenegrin women is nearly the same (43% vs. 41%) [5,6] emphasizing the importance and need for vaccination. The WHO Cervical Cancer Elimination strategy includes coverage targets for scale-up by 2030 of HPV vaccination to 90% of all adolescent girls, twice-lifetime cervical screening to 70%, and treatment of pre-invasive lesions and invasive cancer to 90% [7].

Currently, there are six licensed HPV vaccines available to the population: three bivalent, two quadrivalent, and one nonavalent vaccine. All of these have proven their effectiveness in preventing infection with HPV types 16 and 18, which together cause about 70% of global cervical cancer cases [8,9]. The nonavalent vaccine has the widest prophylactic spectrum, offering additional protection against HPV types 31, 33, 45, 52, and 58, which are responsible for a smaller number of HPV-related cancers. The most common genotypes in Serbian women are HPV 16, 31, 33, and 51 [10]. With this in mind, the prophylactic value of HPV vaccines that target these high-risk strains is evident, and in many countries with implemented HPV vaccination programs and high vaccine coverage, a reduction in the number of HPV infections, genital warts, and cervical precancerous lesions is seen, and reduction in cervical cancer and other HPV related cancers is expected [11,12]. In Serbia, since 2008, the HPV vaccination has been recommended for young adults before their first sexual experience, although the cost of the vaccine was not covered by the national health care insurance. Since June 2022, the nonavalent HPV vaccine was introduced in the National Immunization Program in Serbia and started to be implemented nationwide, targeting persons aged 9–19 years [13]. The vaccine is applied free of charge in two doses for children aged 9–15 and in three doses for young adults aged 15–19 years. In Montenegro, the HPV vaccination campaign started on 26 September 2022, with a one-dose schedule for 9-year-old girls (cohort 2013) as part of the National Immunization Program using the nonavalent HPV vaccine. From 15 February 2023, the catch-up vaccination for all girls aged 9–14 years started (cohorts 2008–2013). Before the start of vaccination, education activities were conducted, especially for healthcare workers at the primary health level (pediatric specialists, pediatric nurses, and gynecologists) and partially among the parents. The program is facility-based and is implemented nationwide. Vaccines are administered by teams that consist of pediatric specialists and pediatric nurses. In Serbia, as well as in Montenegro, immunization against HPV is recommended. During the observed period, the vaccines against HPV were continuously available in both countries.

Lack of parent awareness about vaccination, low level of parental education, gender inequality, and poverty contribute to lower vaccination coverage of children. Similarly, lower education, maternal knowledge, and social prejudices were demonstrated to significantly impact vaccination coverage. Also, various socioeconomic and demographic factors demonstrate a significant relationship with high vaccine coverage, as evidenced by numerous studies conducted at the local and regional levels [14,15,16,17]. The objective of our study was to analyze the coverage of immunization against HPV taking into account available sociodemographic characteristics of different administrative units (districts and municipalities) in Serbia and Montenegro.

## 2. Materials and Methods

### 2.1. Country Characteristics

Both Serbia and Montenegro are divided into administrative units. Observed characteristics of administrative units in our study were: average monthly income, number of pediatricians per 10,000 children, educational attainment of women aged 15 and up (without formal education or incompletely primary education, primary, secondary, and tertiary level of education, or unknown), marital status of women over 15 years old, population density and area (in km^2^) of the district/municipality [18,19]. For the territory of Serbia, the average age of the women aged 15 and over, the degree of urbanization, and the percentage of illiteracy in the population over 10 years old were also observed. In Montenegro, healthcare on the primary health level for 25 administrative units is covered by 18 primary healthcare centers (PHCCs) and seven Health Stations (HSs). HSs are located in small municipalities and administrative parts of some primary healthcare centers.

### 2.2. HPV Vaccine Coverage

We used depersonalized coverage data from the National Immunization Registries of the Public Health Institute of Serbia and Institute of Public Health of Montenegro, which contain data on vaccinated girls with the HPV vaccine. Data were collected for one year from the time of the introduction of nine-valent HPV vaccine at the national level, i.e., from June 2022 in Serbia, and from February 2023 in Montenegro. Additionally, Serbia initiated HPV vaccination, adopting a gender-neutral strategy and targeting ages 9–19 without prior campaign or promotion. In contrast, Montenegro focused initially on 9-year-olds with extensive promotional efforts. It was not until February 2023 that Montenegro expanded its vaccination program to include ages 9–14. We analyzed coverage in cohorts of girls aged 9 to 14 years, 15 to 19, and 9 to 19 years in Serbia, and of girls aged 9–14 years in Montenegro for the mentioned period. The analysis of vaccine coverage was based on the initiated HPV immunization, i.e., every child who received at least one dose of the vaccine was considered vaccinated, in both countries. The coverage by administrative units was calculated by dividing the number of vaccinated female persons by the number of female persons in the observed cohorts according to the population census [20,21].

### 2.3. Data Analyses

Comparisons of categorical data between groups were made by Fisher’s exact test (two-tailed) or chi-square test, where appropriate, while the analysis of variance (ANOVA) was used for continuous data. Tests of proportion were performed in order to compare sociodemographic characteristics and coverage of immunization against HPV between two countries. For the purpose of statistical analyses, different administrative units per country were distributed into tertiles based on the values of the evaluated sociodemographic characteristics, where the first tertile contained the lowest and the third represented the highest values. Pearson’s correlation was used to explore potential correlations between values of different sociodemographic characteristics of the administrative units and the corresponding level of HPV coverage. Data analysis was performed using the SPSS version 22 software and *p* < 0.05 was considered statistically significant across the analyses.

## 3. Results

According to the latest available census data, the total female population aged 9 to 14 is about 188,000 in Serbia and around 25,000 in Montenegro. The average monthly net wage in Montenegro at the beginning of the HPV vaccination program (2022) was USD 776, while in Serbia was USD 670.8. Population density was 79 inhabitants per km^2^ in Serbia and 46 in Montenegro, while the number of pediatricians in primary healthcare was 28.7 per 10,000 children in Serbia and 6.4 in Montenegro. Marital status of females ≥15 years old was represented with the highest percent of married females in both countries, around 53%, while the education of the same population was mostly of secondary level, 48.1% in Serbia and 47% in Montenegro, followed by tertiary (24%) and primary (19.1%) level in Serbia, and primary (22.4%) and tertiary (16.1%) in Montenegro. The average HPV vaccination uptake rate (≥1 dose) of females aged 9–19 years after the first year of immunization in Serbia was 5.72%, (county range across the administrative units 0.92–11.07%, median = 4.80%). On the other hand, in the cohort of females 9–14 years old in Serbia was 5.53%, while in Montenegro was 22.10% for the same age cohort, as presented in Table 1.

**Figure 1:**
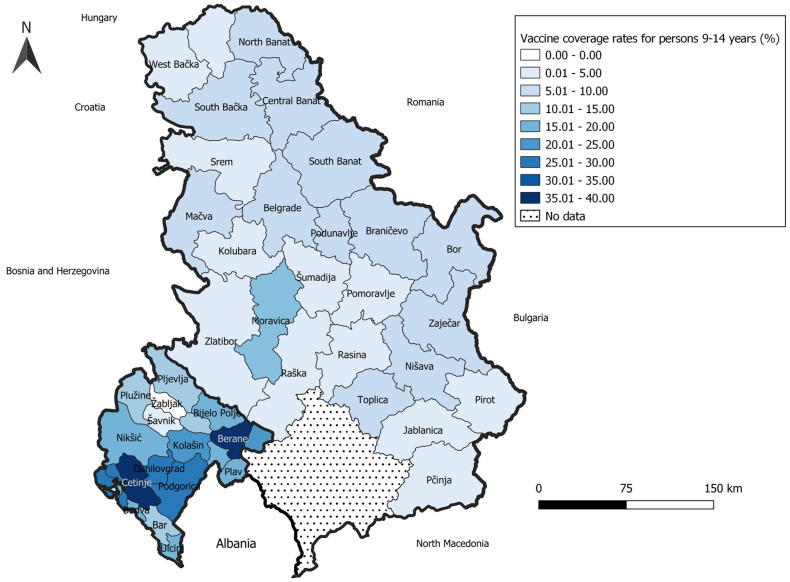
HPV vaccine coverage in girls 9–14 years after first year of immunization across administrative units in Serbia and Montenegro.

Within Serbia, only one administrative region (Moravica) reported HPV immunization coverage in girls 9–19 years old above 10%, 11 districts had coverage from 5 to 10%, while 13 districts of Serbia had coverage below 5%, as presented in Figure 1. On the other hand, the HPV vaccination uptake in Serbia was almost equal between younger females (9–14 years of age) and females aged 15–19 years (5.53% and 5.94%). As per Montenegro, two administrative units, Cetinje and Berane, had the highest coverage, with 39% and 36.4% of vaccinated girls 9–14 years old, respectively. The other four units had coverage between 25 and 30%, another five from 20 to 25%, and the majority was in the range between 15% and 20% of vaccinated eligible girls. Finally, two administrative units, Žabljak and Šavnik administered just a few vaccines and were in the lowest coverage category.

**Figure 2:**
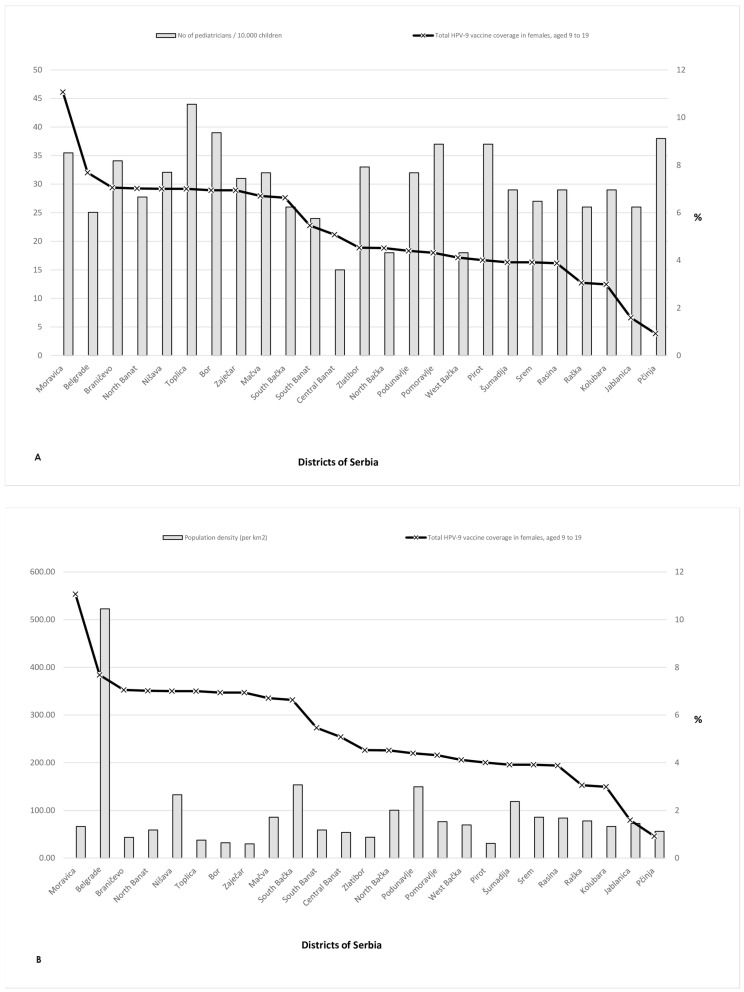
Relation between HPV vaccine coverage in girls 9–19 years old and (**A**) the number of pediatricians in administrative units; (**B**) the district’s population density, across administrative units in Serbia.

When we explored the coverage of HPV immunization among girls aged 9–19 years across different regions in Serbia, ordering coverage from the highest to the lowest, we observed that the level of coverage did not correlate with the number of pediatricians (Figure 2A) or with the population density (Figure 2B). Likewise, the Pčinja region with the lowest vaccination coverage has more pediatricians than the Moravica region, where vaccination coverage is the highest. Also, it can be observed that units with a higher percentage of population density have lower vaccination coverage as we can see in Belgrade and South Bačka, which are more urban than the Moravica region. In the Moravica district, the coverage rate is 11.0%, and in the Pčinja region, 0.9%, while the observed indicator population density is almost equal.

**Figure 3:**
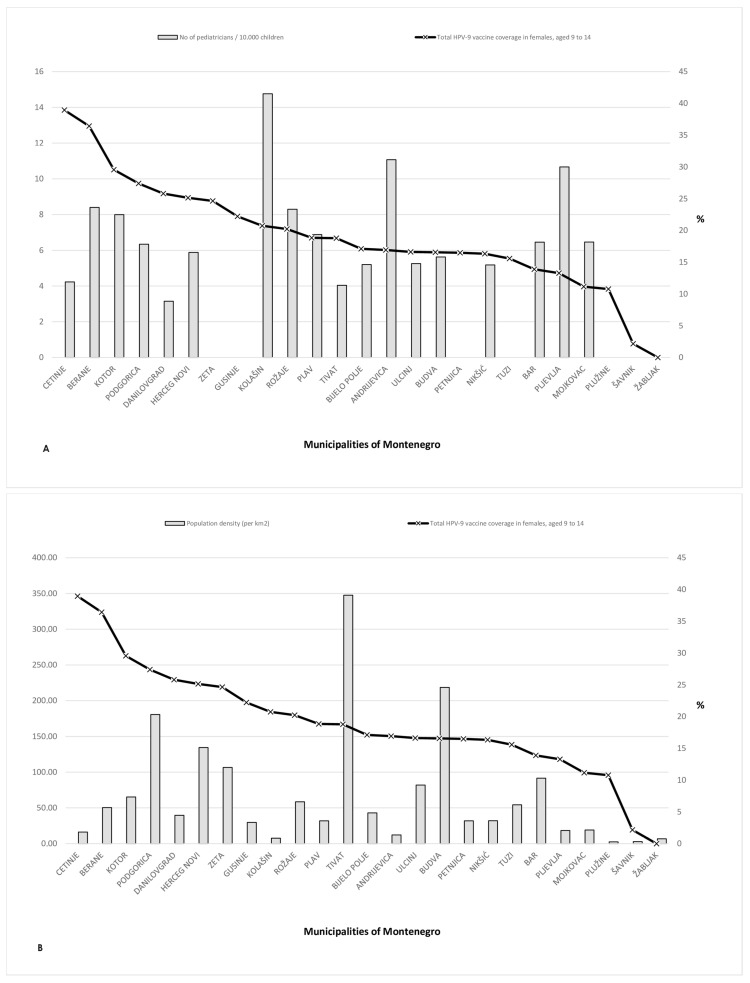
Relation between HPV vaccine coverage in girls 9–14 years old and (**A**) number of pediatricians in administrative units; (**B**) population density, across administrative units in Montenegro.

Vaccine coverage by administrative units in Montenegro (girls 9–14 years), from the highest to the lowest administrative unit is presented in Figure 3. We observed a similar situation as per Serbia’s administrative units, the level of coverage did not correlate with the number of pediatricians or population density. In fact, the administrative units that do not have pediatricians employed in primary healthcare centers (Zeta, Gusinje, Petnjica, Tuzi, Plužine, Šavnik, and Žabljak) have immunization coverage, from 0% in Žabljak to 25% in Zeta region. The administrative unit Cetinje, which has the highest HPV immunization coverage (nearly 40%), counts four employed pediatricians, on the contrary administrative unit Kolašin with 15 employed pediatricians has 20% HPV immunization coverage (Figure 3A). The level of population density (Figure 3B) also did not correlate with the percentage of immunization coverage (Tivat population density 350.0 per km^2^ and coverage below 20%).

When dividing Serbia’s administrative units into tertiles by the distribution of the values of selected sociodemographic variables, we noticed that those units that are in the first tercile (the lowest percentage) by the variable “Illiterate female population aged 10 and up” have the highest mean values of the vaccine coverage (mean = 6.14%, SD = 2.14), although not at the statistically significant level. Administrative units in the third tertile (highest percentage) of the explored variables, “Educational attainment of females aged 15 and up” and “Illiterate female population aged 10 and up”, had the lowest mean values of HPV vaccine coverage (mean 4.33, SD = 2.45 and 4.63, SD = 2.49, respectively). On the other hand, in the administrative units with the highest percentage of married females aged 15 and up (third tertile), the lowest mean value of HPV vaccine coverage has been recorded (mean = 4.00, SD = 2.22) (Table 2).

**Figure 4:**
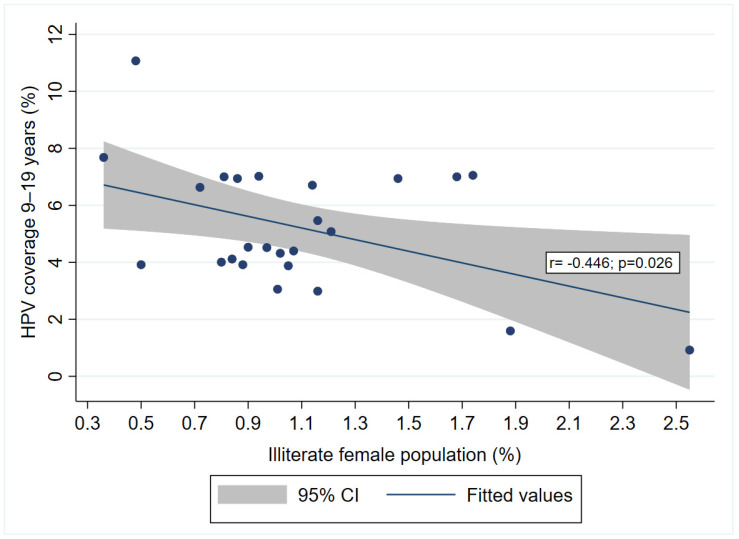
Correlation between administrative units’ percent of illiterate females and the HPV coverage in girls 9–19 yrs old, across districts in Serbia.

Further on, when analyzing a potential correlation between HPV immunization coverage and different sociodemographic characteristics of administrative units in Serbia, we noticed a statistically significant moderate negative correlation (r = −0.446; *p* = 0.026) with the percentage of illiterate women in the administrative units (Figure 4).

In Table 3, we explored the level of vaccine coverage in girls aged 9–14 years across administrative units of Montenegro by terciles of selected sociodemographic variables and observed that the municipalities that are in the level of the second tertile of the observed variable “Married, marital status of females aged 15 and up” reported the highest mean values of coverage (mean 22.77, SD = 8.47) with a statistically significant difference in the level of coverage compared to the first and third tercile (*p* = 0.003). There is also a statistically significant difference in the mean value of the vaccine coverage (*p* = 0.013) across the units from the first tertile (lowest values) of the “Area (in km^2^)” compared to the coverage values in the municipalities that are in the other two terciles (Table 3).

## 4. Discussion

HPV is the most common viral infection in the human reproductive tract, capable of causing cervical cancer in women, other types of cancer, and genital warts in both men and women. By the end of 2022, 130 Member States had incorporated the HPV vaccine into their national immunization programs, including 14 new introductions [11,22]. Despite this progress on the global level, many countries are facing suboptimal vaccine coverage. In 2022, only 21% of girls received the first dose of the HPV vaccine [22]. Challenges in achieving adequate HPV immunization coverage have been observed in various regions. A recent systematic review and meta-analysis, which included 18,611 females from 27 European countries, concluded that personalized interventions targeting population- and country-specific characteristics are necessary to enhance HPV vaccination coverage [23]. However, there exists a dearth of quantitative studies examining the correlation between the socioeconomic characteristics of Eastern and Central European regions, perceptions regarding the severity of specific diseases, and attitudes towards vaccination, particularly in high-income countries within the region, where vaccine hesitancy is most prevalent [22]. Vaccination hesitancy stands as one of the foremost global health threats and has emerged as a critical public health concern worldwide [14].

Although our study only analyzed the first year of vaccination program implementation, we found that the HPV immunization coverage in Serbia was 5.5% for girls aged 9–14 years and 5.9% for those aged 15–19 years. In contrast, Montenegro achieved HPV immunization coverage of 22.1% for girls aged 9–14 years. Despite both countries having high rates of HPV-related cancers, the first-year immunization coverage differed significantly between them [2,8]. Several factors contributed to the differences in immunization coverage between Serbia and Montenegro. The process of introducing the HPV vaccine at the national level differed significantly between the two countries. In Serbia, the HPV vaccine was introduced abruptly, without prior national promotional campaigns, as a gender-neutral vaccination for individuals aged 9–19 and was offered free of charge to those eligible by age [20]. Montenegro had established a structured approach to prepare the target population for the vaccine’s introduction. Conversely, extensive educational campaigns, expert media coverage, school visits, and communication with parents preceded the vaccine’s introduction. This included involving epidemiologists and healthcare workers in promoting and emphasizing the vaccine’s importance to the public. Additionally, financial incentives were provided to those involved in administering the HPV vaccine. These factors, rather than the number of pediatricians, population density, level of education, and other sociodemographic variables investigated in our study, appear to have had a more significant influence on the level of HPV immunization coverage [24].

In Serbia, the national immunization program with the nonavalent HPV vaccine, free of charge for all interested girls and boys aged 9–19, started in June 2022. By the end of June 2023, about 5% of eligible young people had been vaccinated. Although there were no clear reasons for this, the immunization coverage with the nonavalent HPV vaccine in the Moravica District was significantly higher compared to other districts in Serbia. One possible reason could be that epidemiologists from the local Public Health Department had a more prominent presence in local media and communication with schools and healthcare workers in their district compared to other districts. Additionally, immunization coverage against HPV was higher in Montenegro, where there was a smaller population of girls in the appropriate age group for vaccination, making it potentially easier to achieve higher coverage. Furthermore, at the administrative unit’s level, the monthly incomes are higher and population density is lower in Montenegro compared to Serbia, even though the number of pediatricians is inversely proportional to the level of coverage in these two countries. Our research found that the coverage of HPV immunization in Serbia by district was not correlated with the number of pediatricians. The coverage for girls aged 9–14 years was 5.5% in Serbia and 22.1% in Montenegro, indicating that effective organization within the pediatric health service plays a more significant role in implementing HPV immunization than the number of employed pediatricians.

It is noteworthy that in the administrative units in Serbia that fall into the third tertile for the variables “Educational attainment of females aged 15 and up: Without or incomplete primary education” and “Illiterate female population aged 10 and up”, the mean values of average immunization coverage are lower compared to the other tertiles. This suggests that those sociodemographic variables might be relevant to the success of immunization programs. Although the overall HPV coverage is low (5.72%), our study found a correlation between the percentage of illiterate females and the HPV immunization coverage across administrative units in Serbia. This indicates that illiterate individuals might be harder to reach and inform about vaccination.

Considering these findings, along with previously published common reasons for rejecting the HPV vaccine—such as fear of side effects, perception of the vaccine as being too new or experimental, belief that the chances of being infected are low, and concerns regarding safety and inadequate information—it is important to emphasize the significant impact that highly motivated healthcare workers can have on vaccination uptake. Additionally, support from friends or family members, or encouragement from acquaintances, can positively influence vaccination rates against HPV [23]. Considering the prediction that HPV immunization coverage achieved in the first few years tends to be sustained, it is crucial to address these sociodemographic challenges early on. This includes focusing on areas with lower educational attainment and higher illiteracy rates among females, as these factors are significant in achieving successful immunization. Our findings highlight the need for targeted interventions, such as comprehensive educational campaigns and community engagement efforts, particularly in regions with higher illiteracy rates. Additionally, addressing common concerns about the vaccine—like fear of side effects, perceptions of it being new or experimental, and doubts about safety and necessity—can further improve vaccination rates. The most effective preventive measure against human papillomavirus remains vaccination. In Serbia, there is a growing initiative to professionalize educational campaigns involving highly motivated professionals. These campaigns aim to raise awareness among parents and children in schools across every district of the country. Additionally, there is a concerted effort to engage more healthcare workers in educational campaigns tailored for schools in every county. The goal is to ensure that every parent receives crucial information about HPV vaccination directly through the school system. Epidemiologists responsible for each district should have digital information on the number of vaccinated persons, gender, and place of residence, and then analyze vaccination rates more frequently to identify districts with low vaccination coverage. This analysis guides the strengthening of targeted promotional campaigns in those specific areas.

With the advent of the internet, while it brought significant benefits, it also introduced challenges such as the rise of vaccine opponents and their theories. These opposing views have had a considerable impact on public perception regarding vaccination as a crucial preventive measure. It is essential to address these challenges effectively in future national strategies, leveraging media, especially electronic platforms, to counter misinformation and promote evidence-based information about the HPV vaccine. By doing so, we can reinforce the understanding of the vaccine’s importance and encourage higher vaccination rates across communities [25]. These platforms play a pivotal role in disseminating accurate information and effectively reaching a wide audience. In Serbia, utilizing electronic media channels enhances the visibility and understanding of HPV vaccination among the public. This approach ensures that key messages about the vaccine’s benefits and availability are widely accessible, thereby fostering increased vaccination rates nationwide.

Highly motivated healthcare workers play a key role in this process, as do supportive networks involving friends, family, and acquaintances. By leveraging these influences and addressing the specific barriers identified, we can enhance the overall success and sustainability of the HPV immunization programs [26], there is an urgent need to utilize all available resources to increase the uptake of the HPV vaccine in our territory. Considering that the HPV vaccine is proven safe, effective, and provided free of charge for children aged 9–19 years in Serbia, one potential solution could be the implementation of mandatory instead of recommended HPV immunization in our country.

## 5. Conclusions

Despite the fact that the vaccines against HPV were continuously available in both countries, vaccination coverage against HPV in Serbia was low during the first year of the immunization program. One potentially influencing factor might be the literacy levels among the female population at the administrative unit’s level. Drawing on the experiences and successes of HPV immunization in Montenegro, Serbia could improve its vaccination coverage through a robust educational campaign. This campaign should target schools, the general population, and healthcare workers, and include incentives for those involved in these efforts.

## Figures and Tables

**Table 1:** Selected sociodemographic characteristics of Serbia and Montenegro.

	Serbia		Montenegro		*p* Value
Female population, aged 9 to 14 (n)	188,043		24,929		<0.0001 *
Average net monthly income (2022, in USD)	670.8		776		<0.0001 *
Population density (per km^2^)	79		46		0.0054 *
No of pediatrician in primary health care	1012		76		<0.0001 *
No of pediatrician in primary health care (per 10,000 children)	28.67		6.4		NA
Marital status of females aged 15 and up, n (%)					
Unmarried	731,559	22.90%	70,107	27.30%	<0.0001 **
Married	1,702,581	53.40%	13,702	53.40%	
Widow	562,384	17.60%	10,328	4.00%	
Divorced	181,351	5.70%	38,666	15.10%	
Unknown	11,841	0.40%	687	0.30%	
Educational attainment of females aged 15 and up, n (%)					
Without formal education or incomplete primary education	247,375	8.40%	35,993	14.0%	<0.0001 **
Primary education	562,523	19.10%	57,442	22.40%	
Secondary education	1,420,971	48.10%	120,888	47.0%	
Tertiary education	709,436	24.0%	41,278	16.10%	
Unknown	11,507	0.40%	1207	0.50%	
HPV vaccine coverage in the age group 9–14 years (%)	5.53		22.10		NA
HPV vaccine coverage in the age group 15–19 years (%)	5.94		-		NA
HPV vaccine coverage in the age group 9–19 years (%)	5.72		-		NA

* Test of proportion; ** chi-square test. NA—not applicable. In bold are significant results at *p* < 0.05.

**Table 2:** Mean value of HPV-9 vaccine coverage in girls 9–19 years old across administrative units of Serbia by terciles of selected sociodemographic variables.

Administrative Units of Serbia		HPV Vaccine Coverage in Girls 9–19 Years Old		*p*-Value *
		Mean	(SD)	
Degree of Urbanization (2018 Estimate)	1st tertile	4.05	2.07	0.456
	2nd tertile	5.83	2.53	
	3rd tertile	5.96	1.54	
Average net monthly income (2022, in USD)	1st tertile	4.28	2.25	0.248
	2nd tertile	5.62	2.64	
	3rd tertile	5.91	1.35	
	Without or incomplete primary education			
	1st tertile	5.77	2.56	0.157
	2nd tertile	5.53	1.23	
	3rd tertile	4.33	2.45	
	Primary education			
	1st tertile	5.85	2.78	0.116
	2nd tertile	4.68	1.21	
	3rd tertile	5.08	2.3	
	Secondary education			
	1st tertile	4.9	2.21	0.386
	2nd tertile	5.69	1.62	
	3rd tertile	5.15	2.8	
	Tertiary education			
	1st tertile	5.75	1.95	0.302
	2nd tertile	3.59	1.29	
	3rd tertile	6.29	2.39	
Illiterate female population aged 10 and up (2022 census)	1st tertile	6.14	2.42	0.271
	2nd tertile	4.8	1.36	
	3rd tertile	4.63	2.49	
	Unmarried			
	1st tertile	5.39	2.84	0.261
	2nd tertile	5.19	2.22	
	3rd tertile	5.09	1.49	
	Married			
	1st tertile	5.89	1.32	0.138
	2nd tertile	5.69	2.8	
	3rd tertile	4	2.22	
	Widow			
	1st tertile	4.36	2.37	0.56
	2nd tertile	5.89	2.4	
	3rd tertile	5.56	1.63	
	Divorced			
	1st tertile	3.85	2.05	0.281
	2nd tertile	5.84	2.47	
	3rd tertile	6.16	1.3	
Average age of females aged 15 and up (2022 census)	1st tertile	4.74	2.12	0.284
	2nd tertile	5.48	2.91	
	3rd tertile	5.53	1.56	
Population density (per km^2^) by district	1st tertile	5.5	2.11	0.299
	2nd tertile	4.56	2.87	
	3rd tertile	5.6	1.55	
**Area (in km^2^), by district**	1st tertile	4.68	1.39	0.168
	2nd tertile	5.88	2.89	
	3rd tertile	5.19	2.24	
**No of pediatrician/10,000 children**	1st tertile	4.67	1.83	0.257
	2nd tertile	5.36	1.72	
	3rd tertile	5.73	3	
**Number of girls 9–19 yrs old**	1st tertile	5.68	1.64	0.173
	2nd tertile	4.4	3.04	
	3rd tertile	5.55	1.72	

* Using Analysis of variance (ANOVA).

**Table 3:** Mean value of HPV-9 vaccine coverage in girls 9–14 years old across administrative units of Montenegroby terciles of selected sociodemographic variables.

Administrative Units of Montenegro		HPV Vaccine Coverage in Girls 9–14 Years Old		*p*-Value *
		Mean	(SD)	
Marital status of females aged 15 and up (2011 census)	Unmarried			
	1st tertile	20.15	8.18	0.136
	2nd tertile	20.49	5.94	
	3rd tertile	16.03	13.73	
	Married			
	1st tertile	16.93	14.15	**0.003**
	2nd tertile	22.77	8.47	
	3rd tertile	16.96	2.61	
	Widow			
	1st tertile	13.81	6.89	0.567
	2nd tertile	18.54	10.85	
	3rd tertile	24.33	8.68	
	Divorced			
	1st tertile	18.65	4.33	0.054
	2nd tertile	23.33	8.53	
	3rd tertile	14.69	13.04	
Population density (per km^2^) by district	1st tertile	15.13	11.66	0.155
	2nd tertile	20.85	7.1	
	3rd tertile	21.58	5.81	
Area (in km^2^), by district	1st tertile	19.43	3.68	**0.013**
	2nd tertile	19.25	11.44	
	3rd tertile	18.35	11.1	
No of pediatrician/10,000 children	1st tertile	22.27	8.91	0.748
	2nd tertile	18.83	6.35	
	3rd tertile	22.86	8.57	
Number of girls 9–14 yrs old	1st tertile	13.26	7.91	0.947
	2nd tertile	22.9	8.59	
	3rd tertile	21.64	7.57	

* Using ANOVA. In bold = significant *p*-values at <0.05.

## Data Availability

The data that support the findings of this study are available from the corresponding author upon reasonable request.
